# Post-translational control of MAVS signaling: linking mitochondrial antiviral immunity to metabolic and cellular stress contexts

**DOI:** 10.3389/fphys.2026.1907325

**Published:** 2026-08-27

**Authors:** Nao Morimoto, Tomohiko Okazaki

**Affiliations:** 1 Institute for Genetic Medicine, Hokkaido University, Sapporo, Japan; 2 Japan Science and Technology Agency (JST) Fusion Oriented Research for Disruptive Science and Technology (FOREST) Program, Japan Science and Technology Agency (JST), Kawaguchi, Japan; 3 Suntory SunRiSE Fellow, Suntory Foundation of Life Sciences, Kyoto, Japan

**Keywords:** apoptosis, cellular stress, innate immunity, MAVS, mitochondria, post-translational modifications (PTM), type I interferon (IFN-I)

## Abstract

Mitochondria function not only as metabolic and bioenergetic centers but also as critical signaling hubs that integrate cellular context with innate immune response. The mitochondrial antiviral-signaling protein (MAVS), anchored to the outer mitochondrial membrane, is a central adaptor in the RIG-I-like receptor (RLR) pathway, orchestrating type I interferon (IFN) production and apoptosis. Although long regarded as a docking platform for RLR-derived signals, recent advances, particularly concerning its diverse post-translational modifications (PTMs), reveal MAVS as a dynamic integrator that decodes cellular stress and metabolic cues to fine-tune antiviral immunity. Canonical PTMs such as ubiquitination and phosphorylation highlight the importance of precisely controlling both the initiation and downregulation of MAVS signaling, but recent discoveries substantially broaden this regulatory landscape. Stress-responsive phosphorylation mediated via the ASK1–p38 MAPK pathway enhances MAVS signaling capacity under oxidative and ER stress, linking cellular damage to amplified interferon production. In parallel, a newly identified vitamin K–dependent carboxylation of MAVS reshapes downstream signaling by promoting interferon induction while restraining apoptosis, introducing a regulatory layer that may reflect the metabolic context surrounding GGCX activity, including vitamin K availability. Understanding this multilayered regulatory network not only redefines MAVS as a stress-sensitive mitochondrial signaling hub responsive to cellular context but also highlights new avenues for therapeutic modulation of innate immunity and cell fate during viral infection. This review summarizes emerging insights into PTM-mediated regulation of MAVS and outlines their broader implications for mitochondrial antiviral signaling.

## 1 Introduction: mitochondria as hubs for antiviral and stress signaling

Mitochondria are classically recognized as central organelles for cellular bioenergetics and have also been established as key hubs for innate immune signaling (Weinberg et al., 2015). In antiviral defense, mitochondria-associated signaling pathways regulate early host responses, including type I interferon (IFN) production and apoptosis, to restrict viral replication and maintain cellular homeostasis (Okazaki, 2017; Stetson and Medzhitov, 2006). At the same time, mitochondria are increasingly appreciated as dynamic organelles that respond to metabolic and stress-related cellular contexts such as oxidative stress, endoplasmic reticulum (ER) stress, heat shock, and DNA damage (Palmer, 2022; Foo et al., 2022; Quirós et al., 2016). These considerations highlight the importance of examining mitochondrial antiviral responses in connection with the metabolic and stress-related cellular contexts.

Upon viral infection, RIG-I-like receptors (RLRs), including RIG-I and MDA5, recognize viral RNA in the cytosol (Yoneyama et al., 2004; Gitlin et al., 2006). Activated RLRs then interact with the common adaptor protein mitochondrial antiviral-signaling protein (MAVS, also known as IPS-1, VISA, or CARDIF). MAVS is a tail-anchored adaptor protein primarily localized to the outer mitochondrial membrane, with additional localization reported on peroxisomes and mitochondria-associated ER membranes (MAMs) (Kawai et al., 2005; Seth et al., 2005; Meylan et al., 2005; Xu et al., 2005; Dixit et al., 2010; Horner et al., 2011). Following MAVS activation, downstream signaling branches into multiple outputs, including type I IFN induction, inflammatory signaling, and apoptosis (Besch et al., 2009). Because these outputs are functionally distinct, their relative engagement is likely subject to diverse regulatory mechanisms.

Among these mechanisms, post-translational modifications (PTMs) of MAVS represent an important layer of control over MAVS function. Early studies mainly focused on ubiquitination and phosphorylation as canonical mechanisms controlling MAVS activation and termination. Subsequent work has expanded this view by revealing that diverse PTMs modulate MAVS signaling capacity, stability, and functional output. Together, these findings have contributed to a shift in the view of MAVS from a docking platform for RLR-mediated signaling to a dynamically regulated signaling hub that fine-tunes antiviral responses in a context-dependent manner.

In this review, we summarize the current understanding of PTM-mediated regulation of MAVS and discuss how these modifications influence both the magnitude and the qualitative nature of antiviral signaling, with particular attention to their potential links to metabolic and stress-related cellular contexts.

## 2 MAVS signaling: canonical antiviral pathways

### 2.1 Domain organization of MAVS

**Figure 1:**
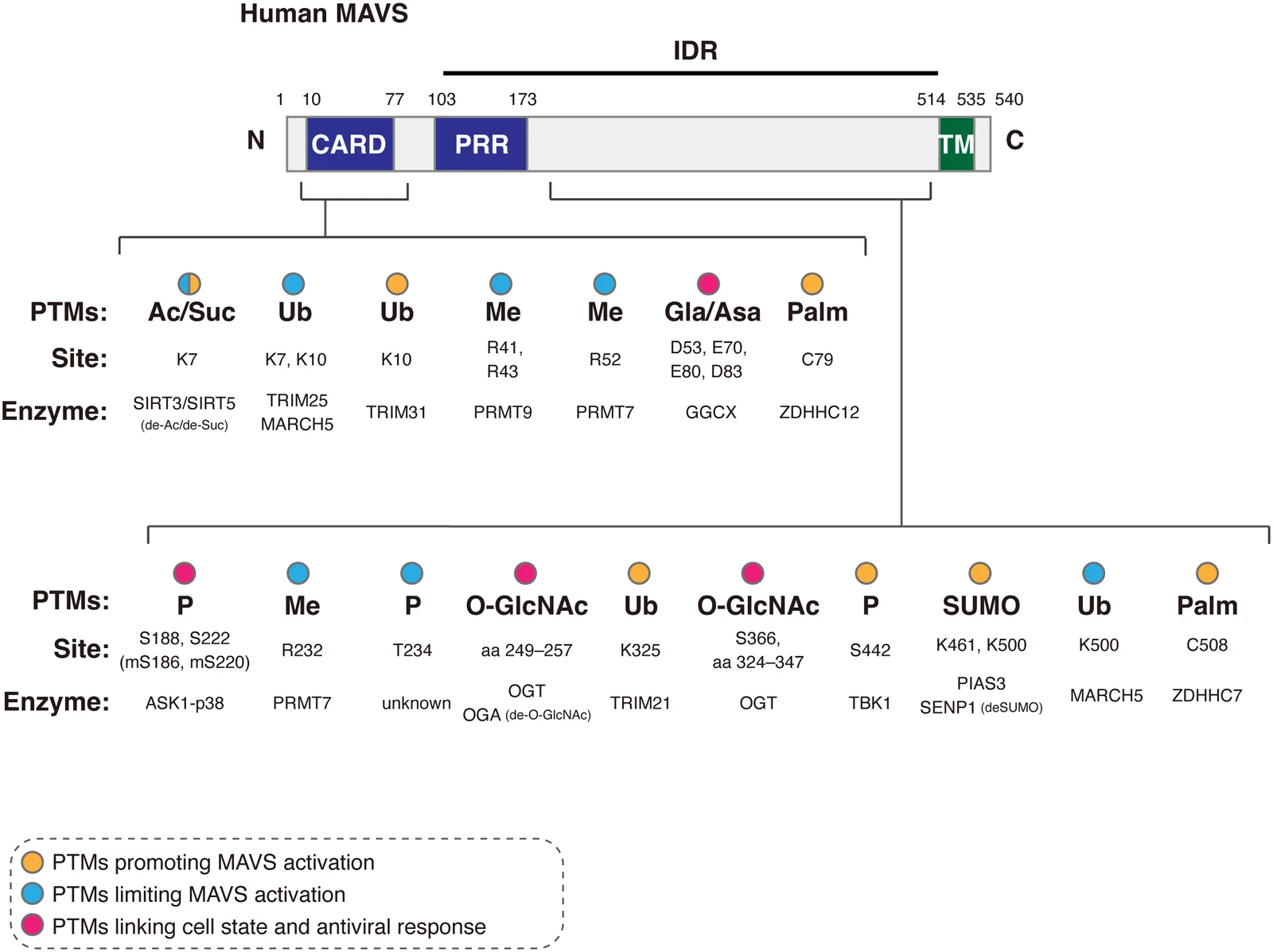
Domain organization and post-translational modification (PTM) landscape of human MAVS. Human MAVS consists of an N-terminal CARD (residues 10-77), a PRR (103-173), an intrinsically disordered region (IDR), and a C-terminal TM domain (514–535). The two rows beneath the MAVS domain map summarize representative post-translational modifications (PTMs), including acetylation and succinylation (Ac/Suc), ubiquitination (Ub), methylation (Me), γ-carboxylation (Gla), β-carboxylation (Asa), phosphorylation (P), O-GlcNAcylation (O-GlcNAc/de-O-GlcNAc), SUMOylation (SUMO/deSUMO), and palmitoylation (Palm). Each PTM is mapped to its corresponding modification sites and regulatory enzymes. The split-color symbol for Ac/Suc reflects the opposing regulatory effects associated with acetylation and succinylation at K7. S188 and S222 refer to human MAVS numbering and correspond to mouse S186 (mS186) and S220 (mS220), respectively. aa, amino acid residues.

To understand how PTMs regulate MAVS signaling, it is important to consider the domain organization of MAVS, as the functional consequences of PTMs are strongly influenced by their structural context. Human MAVS is a 540-amino-acid protein composed of multiple functional regions that coordinate antiviral signaling (Seth et al., 2005) (**Figure 1**).

At the N-terminus, the caspase activation and recruitment domain (CARD) mediates homotypic CARD-CARD interactions with activated RIG-I and MDA5, initiating MAVS activation during viral infection (Chen et al., 2021).

Adjacent to the CARD, MAVS contains a broad central intrinsically disordered region (IDR) that includes the proline-rich region (PRR) (van der Lee et al., 2014) (**Figure 1**). This IDR contains multiple regulatory phosphorylation sites, while the PRR harbors TNF receptor-associated factor (TRAF)-binding motifs that mediate interactions with TRAF family adaptor proteins, including TRAF2, TRAF3, TRAF5, and TRAF6 (Xu et al., 2005; Saha et al., 2006; Tang and Wang, 2010). More recent work further showed that the MAVS IDR directly binds cellular RNAs, particularly the 3’ untranslated regions (UTRs) of host mRNAs, thereby modulating MAVS protein-protein interactions, signalosome assembly, and interferon induction (Gokhale et al., 2024). These features support the view that the MAVS IDR functions as a flexible regulatory interface for coordinating MAVS-dependent antiviral signaling.

Finally, the C-terminal transmembrane (TM) domain anchors MAVS to the mitochondrial outer membrane, and this mitochondrial targeting is required for efficient MAVS-dependent signaling (Seth et al., 2005).

Together, these domain features indicate that MAVS PTMs should be interpreted in relation to their positions within the protein, as modifications in distinct regions are likely to have different functional consequences.

### 2.2 Canonical MAVS activation and signalosome formation

**Figure 2:**
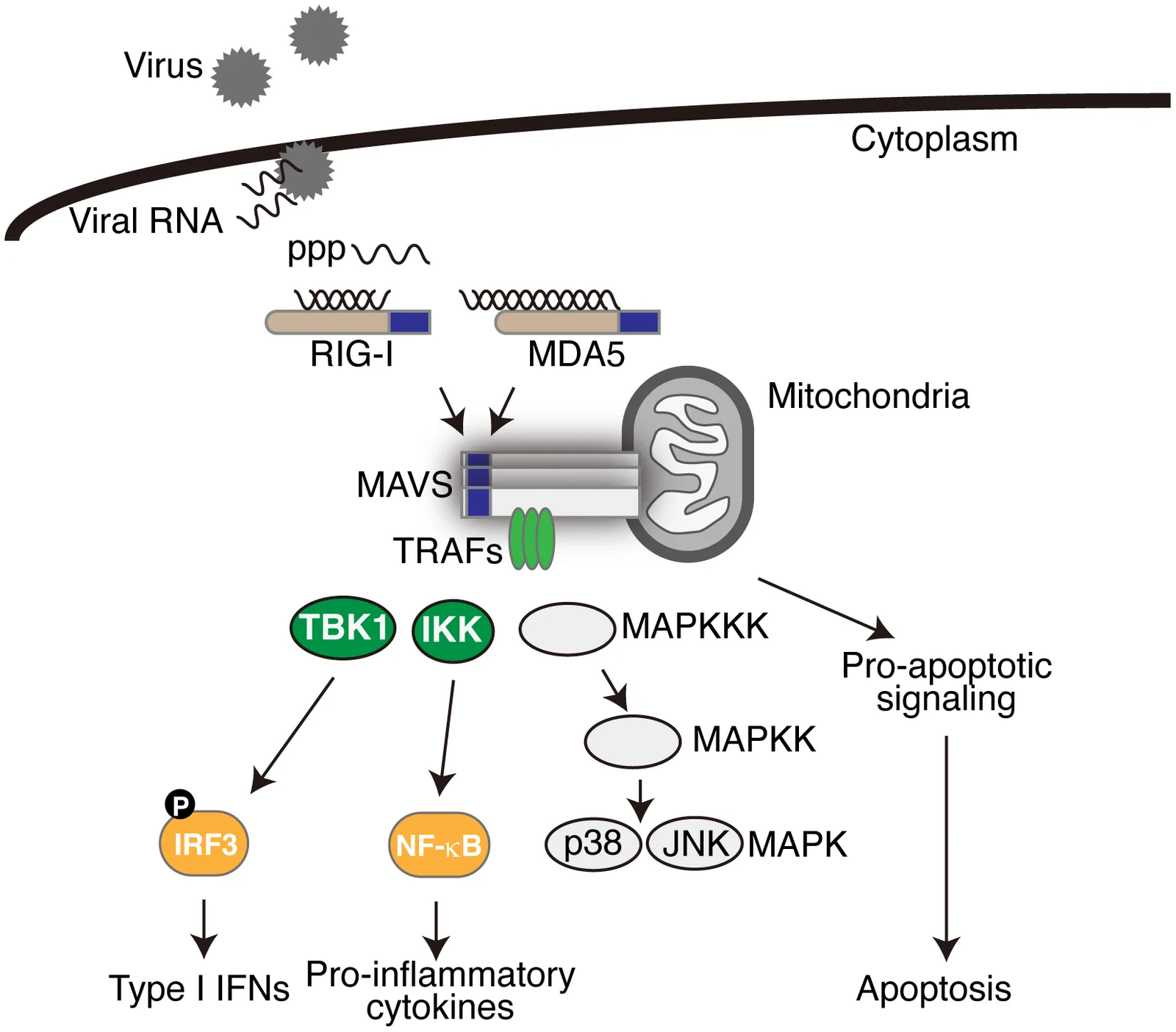
The RLR-MAVS signaling pathway in antiviral innate immunity. Viral infection leads to the release of viral RNA into the cytoplasm, where it is recognized by the RIG-I-like receptors (RLRs), RIG-I and MDA5. Upon sensing viral RNA, activated RLRs interact with MAVS on the outer mitochondrial membrane, leading to MAVS activation and the recruitment of TRAF family adaptor proteins. MAVS-associated TRAFs promote the activation of TBK1, the IKK complex, and MAPK signaling pathways. In the MAPK branch, sequential activation of MAPKKKs and MAPKKs leads to activation of downstream MAPKs, including p38 and JNK. These signaling pathways induce type I IFN production and pro-inflammatory cytokine production. In addition to these responses, MAVS-dependent signaling triggers pro-apoptotic pathways, ultimately leading to host cell apoptosis as a defense mechanism against viral infection.

Following RLR engagement, MAVS assembles into higher-order, prion-like signaling complexes known as MAVS signalosomes (F. Hou et al., 2011). These membrane-associated assemblies support downstream signal propagation through TRAF-dependent activation of downstream kinases, including TBK1 and the IKK complex. Activation of these kinases leads to interferon regulatory factors 3 and 7 (IRF3/7) and nuclear factor-kappa B (NF-κB) activation, resulting in the induction of type I interferons (IFNs) and pro-inflammatory cytokines (Konno et al., 2009; S. Liu et al., 2013; Meylan et al., 2005; Xu et al., 2005; Saha et al., 2006). MAVS signaling also engages mitogen-activated protein kinase (MAPK) pathways, including p38 and c-Jun N-terminal kinase (JNK), further contributing to antiviral and inflammatory responses (Refolo et al., 2020; Vazquez and Horner, 2015) (**Figure 2**).

In addition to these interferon- and inflammatory signaling pathways, MAVS can also trigger pro-apoptotic programs. Building on this overview of MAVS signaling, the following section discusses how major PTMs, particularly ubiquitination and phosphorylation, regulate MAVS activation and signal propagation.

## 3 PTM-mediated regulation of the MAVS signaling cycle

MAVS PTMs can be broadly grouped into modifications that promote antiviral signal propagation and those that restrain signaling to restore homeostasis (Ren et al., 2020; Dong and Shen, 2024). Rather than revisiting the full spectrum of MAVS PTMs covered in excellent previous reviews (Ren et al., 2020; Dong and Shen, 2024; Chen et al., 2021), this section highlights representative MAVS PTMs and recent advances that illustrate how MAVS signaling is regulated at distinct functional layers, including MAVS quiescence, signalosome assembly, downstream branch activation, and signal attenuation. Later in this review, we discuss how cellular metabolic and stress contexts may further shape MAVS signaling.

### 3.1 PTMs maintaining MAVS quiescence

Arginine methylation contributes to basal-state regulation of MAVS. PRMT7-mediated methylation at R52 and R232 attenuates MAVS activation by limiting its interaction with RIG-I and TRIM31, whereas PRMT9-mediated methylation at R41 and R43 helps prevent spontaneous MAVS aggregation in resting cells, thereby supporting innate immune homeostasis (Bai et al., 2022; Zhu et al., 2021; Yang et al., 2024).

### 3.2 PTMs regulating MAVS activation and signalosome assembly

Several PTMs enhance MAVS activation by promoting MAVS aggregation, stabilizing signaling assemblies, or facilitating the recruitment of downstream signaling factors.

K63-linked ubiquitination mediated by TRIM31 contributes to the formation of higher-order MAVS aggregates and the amplification of antiviral signaling (B. Liu et al., 2017). Although TRIM31 catalyzes K63-linked ubiquitination of MAVS even under uninfected conditions, MAVS aggregation occurs only following virus-induced RIG-I activation, suggesting that this modification establishes a permissive state for subsequent MAVS aggregation. MAVS assembly is also promoted by PIAS3-mediated poly-SUMOylation, which provides a distinct mechanism for organizing the signaling complex. Poly-SUMO chains attached to MAVS engage a SUMO-interacting motif (SIM) within MAVS, thereby generating multivalent interactions that drive the formation of liquid-like MAVS condensates (Dai et al., 2023). These condensates enrich IRF3 through its own SIM, increasing its local concentration within the MAVS signaling complex and promoting downstream antiviral signaling. In addition to supporting condensate formation, PIAS3-mediated SUMOylation enhances MAVS K63-linked ubiquitination and aggregation, suggesting that SUMOylation can facilitate ubiquitin-dependent MAVS signalosome assembly (Dai et al., 2023). This aggregation-promoting activity of SUMOylation is opposed by regulated deSUMOylation. TOLLIP binds the intrinsically disordered region of MAVS and recruits the SUMO protease SENP1, thereby promoting MAVS deSUMOylation (J. Hou et al., 2025).

Alternative acylation states provide an additional layer of regulation at the level of MAVS assembly. At lysine 7, MAVS can undergo either acetylation or succinylation. Although the enzymes that install these modifications remain unidentified, they are removed by SIRT3 and SIRT5, respectively. SIRT3-mediated deacetylation promotes MAVS aggregation and downstream TBK1-IRF3 signaling, whereas SIRT5-mediated desuccinylation suppresses MAVS aggregation and type I interferon responses. Acetylation and succinylation at this shared residue appear to interfere with one another during viral infection (X. Liu et al., 2024). Notably, lysine 7 is also subject to K48-linked ubiquitination (Castanier et al., 2012; Yoo et al., 2015; Dong and Shen, 2024), illustrating how distinct modifications at a single site can differentially regulate MAVS signaling.

Palmitoylation has also been implicated in MAVS aggregate formation and stabilization. Following K63-linked ubiquitination, ZDHHC12-mediated palmitoylation at cysteine 79 functions as a checkpoint required for virus-induced MAVS aggregation (L. Wang et al., 2024). A distinct palmitoylation site, cysteine 508, is located adjacent to the C-terminal transmembrane domain, raising the possibility that its modification might regulate MAVS membrane targeting. However, ZDHHC7-mediated palmitoylation at this site is dispensable for basal mitochondrial localization and instead stabilizes MAVS aggregation on the mitochondrial outer membrane, thereby promoting antiviral signaling (Y. Liu et al., 2024).

### 3.3 PTMs controlling downstream branch activation and signal attenuation

In addition to PTMs that promote or stabilize MAVS assembly, other modifications enhance MAVS signaling by strengthening interactions with downstream signaling factors. TRIM21 catalyzes K27-linked ubiquitination of MAVS at lysine 325 within the central intrinsically disordered region, enhancing the interaction between MAVS and TBK1. TRIM21 depletion also reduces virus-induced expression of type I and type III interferons, interferon-stimulated genes, and pro-inflammatory cytokines, indicating that TRIM21-dependent MAVS regulation supports multiple antiviral and inflammatory outputs (Xue et al., 2018). TBK1-mediated phosphorylation of MAVS at a C-terminal serine cluster promotes IRF3 recruitment and activation, thereby linking MAVS activation to transcriptional induction of antiviral genes (S. Liu et al., 2015).

MAVS signaling can also be restrained by mechanisms that interfere with downstream signal propagation. Phosphorylation of MAVS at threonine 234, mediated by an as-yet-unidentified kinase, may provide a permissive docking site for PLK1. MAVS-associated PLK1 inhibits TRAF3 recruitment and suppresses signaling through both the IRF3 and NF-κB pathways, thereby limiting antiviral and inflammatory gene induction (Vitour et al., 2009).

Finally, K48-linked ubiquitination provides a degradation-based mechanism for terminating MAVS signaling. Multiple E3 ubiquitin ligases, including TRIM25 and MARCH5, target MAVS for proteasomal degradation, thereby contributing to signal termination (Castanier et al., 2012; Yoo et al., 2015; Dong and Shen, 2024).

### 3.4 Crosstalk among MAVS PTMs and signaling outputs

MAVS PTMs do not appear to function as isolated regulatory events. Rather, they can act sequentially, reinforce or oppose one another, and converge on shared residues, thereby shaping the magnitude and duration of MAVS signaling.

A limited number of individual MAVS PTMs discussed in the following sections have been shown to exert output-selective effects. However, relatively few studies have directly compared IRF3-dependent interferon induction, NF-κB-dependent inflammatory responses, and MAVS-dependent apoptosis within the same experimental setting. Consequently, whether crosstalk among distinct PTMs also contributes to signaling-output selection, beyond regulating the overall magnitude and duration of MAVS activation, remains unclear.

## 4 MAVS as a hub for metabolic and stress-responsive regulation in antiviral immunity

Cellular metabolic and stress responses are increasingly recognized to intersect with innate immune signaling (Muralidharan and Mandrekar, 2013; O’Neill and Pearce, 2016). Recent studies of MAVS PTMs have begun to reveal how this intersection is implemented at the level of MAVS regulation. In this section, we discuss two examples of such context-dependent regulation: O-GlcNAcylation, which links cellular metabolic status to MAVS signaling, and ASK1–p38-dependent phosphorylation, which connects cellular stress pathways to MAVS regulation.

O-GlcNAcylation is a PTM that integrates cellular metabolic status into innate immune signaling (Hart, 2014; Y. Wang et al., 2023). UDP-GlcNAc, the substrate for O-GlcNAcylation, is produced through the hexosamine biosynthetic pathway (HBP).

**Figure 3:**
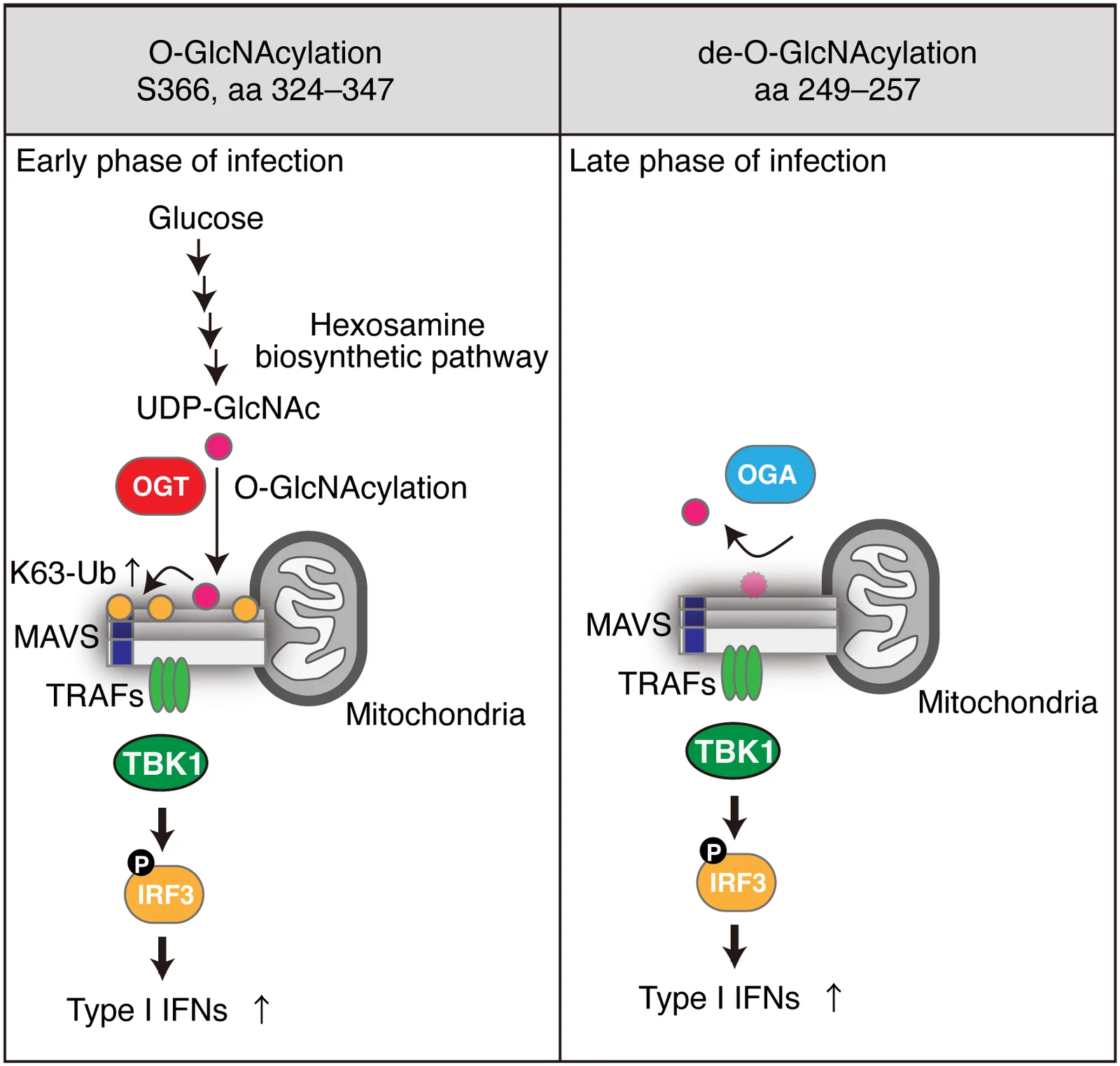
Spatiotemporal regulation of MAVS activity by O-GlcNAcylation during viral infection. In the early phase of infection (left panel), increased glucose metabolism through the hexosamine biosynthetic pathway (HBP) elevates UDP-GlcNAc levels. O-GlcNAc transferase (OGT) then mediates the O-GlcNAcylation of MAVS at S366 and multiple residues within the aa 324–347 region. This modification promotes MAVS K63-linked ubiquitination (Ub), thereby enhancing TRAF recruitment and subsequent TBK1–IRF3 signaling, leading to robust type I IFN production. In the late phase of infection (right panel), O-GlcNAcase (OGA) catalyzes de-O-GlcNAcylation within the aa 249–257 region. Removal of the O-GlcNAc moiety promotes MAVS aggregation and TRAF3-dependent signaling, thereby supporting IFN-β production and contributing to a balanced antiviral response as the infection progresses. aa, amino acid residues.

Recent studies suggest that O-GlcNAcylation of MAVS is regulated in a cell type– and site-dependent manner, producing distinct regulatory outcomes in macrophages and epithelial cells, as discussed by Seo et al. (Seo et al., 2020) (**Figure 3**). In macrophages, viral infection in the early phase is associated with increased glucose metabolism and elevated expression of O-GlcNAc transferase (OGT), a key downstream effector of the hexosamine biosynthetic pathway, leading to O-GlcNAcylation of MAVS at S366 and multiple residues within the 324–347 aa region. This addition of O-GlcNAc promotes K63-linked ubiquitination of MAVS and enhances IFN responses (Song et al., 2019; Li et al., 2018). Notably, flux through the hexosamine biosynthetic pathway itself appears to be required for efficient induction of antiviral signaling, as suggested by pharmacological inhibition of this pathway (Song et al., 2019).

In contrast, in epithelial cells, MAVS O-GlcNAc modification has been mapped to a region of MAVS (aa 249–257) distinct from the sites identified in macrophages. During the late phase of infection, OGT expression decreases and cellular O-GlcNAcylation is globally reduced. Inhibition of O-GlcNAcase (OGA) suppresses MAVS-dependent IFN-β induction, suggesting that removal of O-GlcNAc from this region facilitates MAVS aggregation, interaction with TRAF3, and IFN-β production (Seo et al., 2020). Thus, whereas O-GlcNAc addition promotes MAVS activation in macrophages, O-GlcNAc removal appears to support MAVS activation in epithelial cells, highlighting the cell type- and site-dependent nature of this modification. The physiological significance of this cell type-dependent divergence remains an open question. In particular, future studies will be needed to determine how cell type-specific metabolic states shape MAVS O-GlcNAcylation and its functional consequences during antiviral responses.

**Figure 4:**
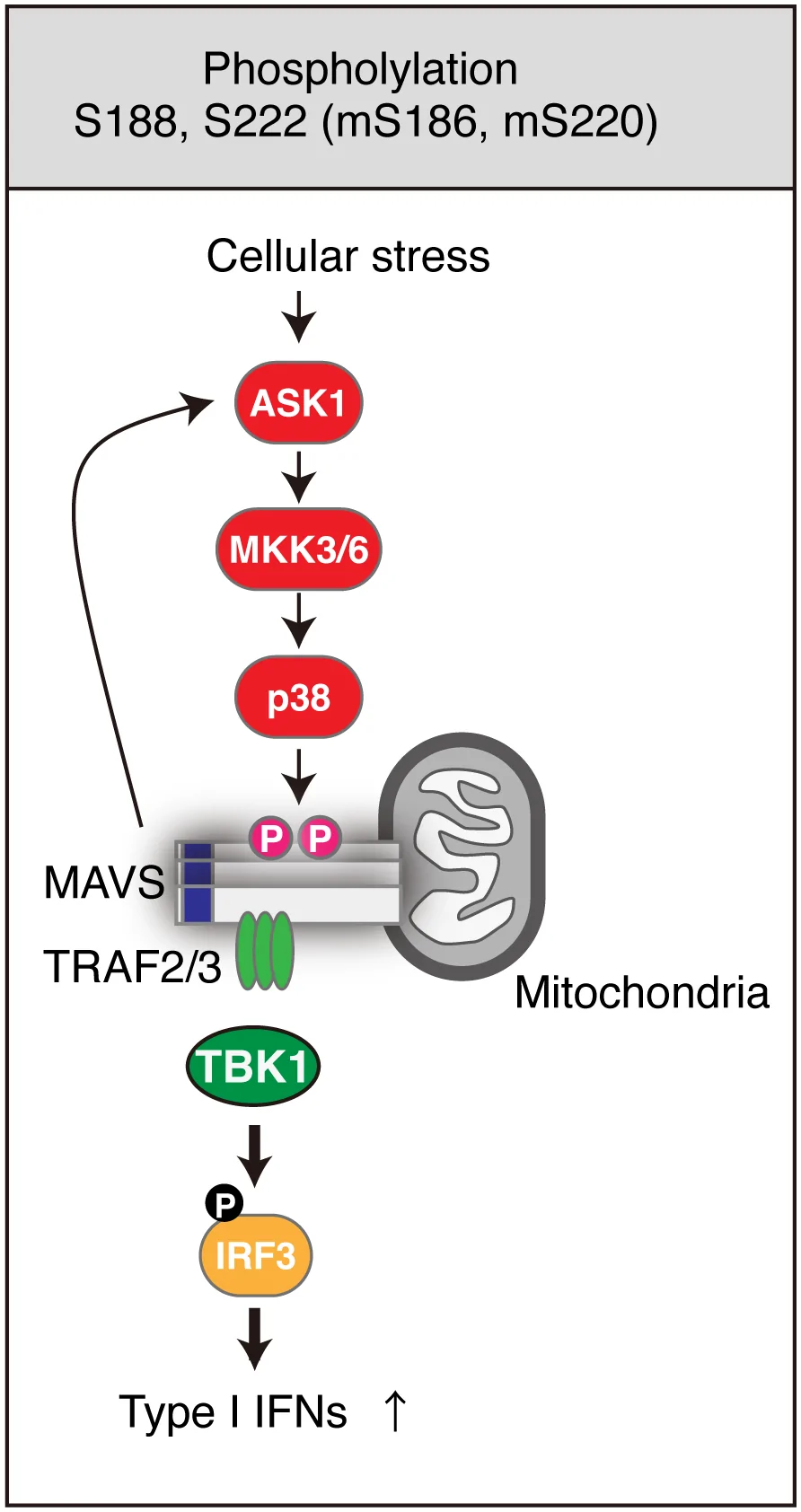
Stress-induced phosphorylation of MAVS via the ASK1–p38 pathway. Various cellular stresses trigger the activation of the mitogen-activated protein kinase (MAPK) cascade, involving ASK1 and MKK3/6, leading to the subsequent activation of p38 MAPK. Activated p38 mediates the phosphorylation of MAVS at specific serine residues, including S188 and S222 (corresponding to mS186 and mS220 in mice). This phosphorylation event enhances the recruitment of TRAFs and the activation of IRF3, promoting the induction of type I IFNs.

In addition to metabolic regulation, cellular stress pathways have also been shown to modulate MAVS signaling through MAVS phosphorylation (Zhao et al., 2025). Oxidative and endoplasmic reticulum (ER) stress activate the stress-responsive MAPKKK ASK1, which in turn engages the p38 MAPK pathway, leading to phosphorylation of mouse MAVS at S186 and S220, which correspond to human S188 and S222, respectively (**Figure 4**). This modification enhances the interaction of MAVS with TRAF family adaptors (including TRAF2 and TRAF3) and promotes type I IFN induction during viral infection. Notably, this phosphorylation is essential for robust induction of type I IFNs in response to RNA virus infection, but is dispensable for MAVS-mediated apoptotic signaling, suggesting a specific role in fine-tuning antiviral innate immunity. Together, these findings highlight that stress-activated kinase cascades function as key modulators of MAVS-dependent antiviral responses. Interestingly, reanalysis of phosphoproteomic datasets from insulin-stimulated cells has revealed increased phosphorylation of MAVS at S186 and S220 (Zhao et al., 2025; Humphrey et al., 2013; Parker et al., 2015), suggesting that MAVS phosphorylation at these residues may represent a shared regulatory point at which stress-related and metabolic signaling inputs converge on MAVS. In this context, given that ASK1 is known to be activated downstream of MAVS (Okazaki et al., 2015), phosphorylation of MAVS may serve as a critical node in a positive feedback loop that amplifies MAVS-dependent type I IFN production, particularly during the early stages of infection. Such a mechanism may allow stress-dependent MAVS phosphorylation to enhance antiviral IFN responses without increasing apoptotic signaling.

## 5 Cytoplasmic carboxylation of MAVS: a determinant of signaling fate

MAVS functions as a central signaling adaptor that coordinates both type I IFN production and apoptosis during viral infection; however, how the balance between these two distinct antiviral outputs is regulated remains largely unclear.

**Figure 5:**
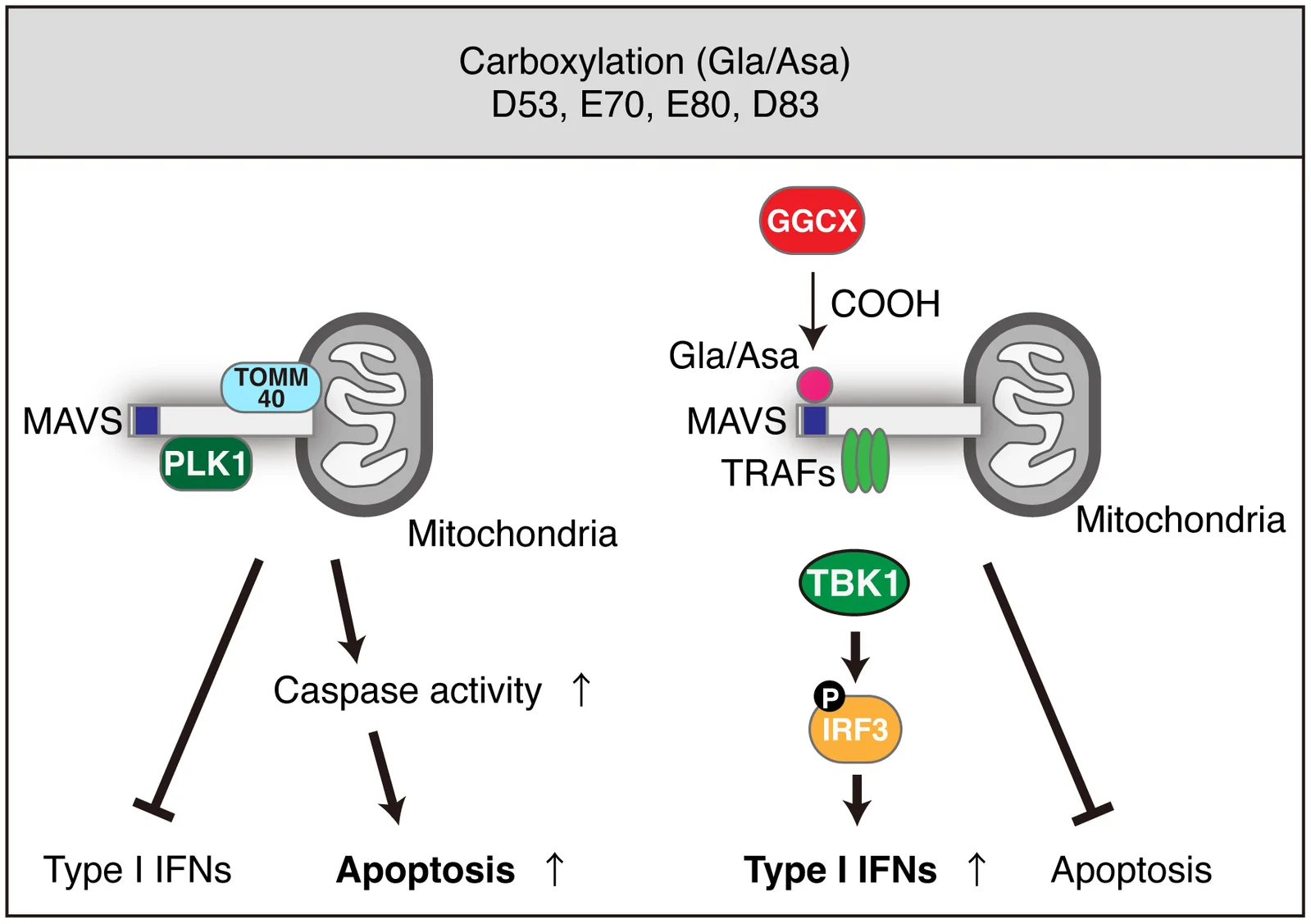
Regulation of MAVS-mediated antiviral responses and apoptosis by vitamin K-dependent carboxylation. The left panel illustrates the signaling state in the absence of MAVS carboxylation. MAVS shows increased association with TOMM40 and PLK1, and this state is associated with reduced type I IFN production and increased caspase activity, thereby favoring apoptosis over IFN-mediated antiviral signaling. The right panel shows the regulatory effect of carboxylation mediated by the enzyme GGCX at residues D53, E70, E80, and D83. The addition of carboxyl groups (-COOH) to MAVS promotes IRF3 activation, thereby increasing type I IFN production. Conversely, GGCX-mediated carboxylation inhibits pro-apoptotic signaling, thereby prioritizing the IFN response over cell death during viral challenge.

Recent work has identified vitamin K–dependent carboxylation of MAVS as a previously unrecognized cytoplasmic PTM, with modification sites mapped to Asp53, Glu70, Glu80, and Asp83 (Okazaki et al., 2025) (**Figure 5**). This modification comprises β-carboxylation (Asa) at the Asp residues and γ-carboxylation (Gla) at the Glu residues. Although γ-glutamyl carboxylase (GGCX) is conventionally viewed as an ER-resident enzyme with its catalytic domain facing the ER lumen (Tie and Stafford, 2016), activation of the ASK1–MKK6–p38 signaling pathway during viral infection causes GGCX to undergo topology inversion. This topological change places the catalytic domain of GGCX on the cytoplasmic side of the ER membrane, thereby enabling GGCX to physically access and carboxylate MAVS (Okazaki et al., 2025). Strikingly, this modification enhances IFN induction while concomitantly suppressing apoptosis. This dual effect indicates that carboxylation selectively biases MAVS signaling toward IFN-mediated antiviral responses. In this regard, MAVS carboxylation may function as a molecular switch that shapes the qualitative nature of antiviral immunity rather than merely modulating signaling magnitude. Given that this modification is mediated by vitamin K-dependent GGCX, this regulatory mechanism may provide a potential conceptual link between MAVS signaling fate and the metabolic context surrounding GGCX activity, including vitamin K availability. The physiological relevance of this carboxylation-dependent switch is supported by *in vivo* studies. Neural cell–specific ablation of Ggcx reduced survival after VSV infection in the brain, accompanied by increased brain viral titers, reduced IFN-α/β induction, and enhanced caspase activation, suggesting a shift of MAVS-mediated responses away from type I IFN and toward apoptosis. Notably, the selective impact in neural cells suggests that this switch may be particularly relevant in tissues with limited regenerative capacity, where restraining apoptosis while sustaining IFN responses could help preserve tissue integrity during infection.

Proteomic identification of MAVS-interacting proteins revealed that TOMM40 and PLK1 preferentially associate with a carboxylation-deficient MAVS mutant (Asp53Ala, Glu70Ala, Glu80Ala, and Asp83Ala) compared with wild-type MAVS. Functionally, TOMM40 enhanced MAVS-mediated caspase activation (Okazaki et al., 2025). For PLK1, previous work has shown that it negatively regulates MAVS-dependent IFN-β induction and that its recruitment is supported by MAVS Thr234 phosphorylation (Vitour et al., 2009). Thus, the enrichment of PLK1 on carboxylation-deficient MAVS raises the possibility that MAVS carboxylation influences PLK1–MAVS regulation, including PLK1 recruitment linked to Thr234 phosphorylation. Together, these findings indicate that modification-dependent remodeling of the MAVS interactome may underlie the switch between apoptotic and IFN-dominant antiviral responses.

## 6 Perspectives: context-dependent regulation of MAVS signaling

As discussed throughout this review, MAVS activity is regulated by a wide range of PTMs that operate at multiple regulatory layers. These PTMs do not merely control the amplification or termination of antiviral signaling. Rather, they enable MAVS to integrate diverse intracellular cues, including metabolic states and cellular stress, thereby shaping context-appropriate outputs that contribute to the maintenance of cellular homeostasis. In this regard, mitochondrial localization may be particularly important, as it places MAVS signaling at an organelle where metabolic regulation, stress responses, and apoptosis are closely interconnected (Palmer, 2022; Foo et al., 2022; Quirós et al., 2016). MAVS localization at mitochondria may therefore provide a spatial basis for tuning MAVS signaling according to the state of the organelle.

Consistent with this view, previous studies have suggested that altering the mitochondrial targeting of MAVS can preserve IFN-inducing activity while selectively impairing apoptotic signaling, highlighting the particular sensitivity of MAVS-dependent apoptosis to mitochondrial localization (Okazaki et al., 2013). This output-specific dependence on mitochondrial localization raises the broader question of how subcellular localization and regulatory PTMs cooperate to shape MAVS signaling outputs. A notable example of localization-dependent PTM function is provided by MAVS palmitoylation. Recent work demonstrates that MAVS palmitoylation is dispensable for basal mitochondrial localization but is required for the accumulation and aggregation of activated MAVS in the mitochondrial pool, whereas a comparable effect was not observed for MAM-localized MAVS (Y. Liu et al., 2024). Additional evidence for compartment-linked regulation comes from Gp78/AMFR, a MAM-associated E3 ubiquitin ligase reported to reduce MAVS protein abundance and attenuate MAVS signaling (Jacobs et al., 2014). Together, these findings suggest that the functional consequences of at least some PTMs depend on organelle context and raise the possibility that organelle-specific MAVS pools differ not only in their intrinsic signaling outputs but also in their responsiveness to regulatory PTMs. Whether other MAVS PTMs also exhibit organelle-specific regulation remains largely unexplored, as most studies have not distinguished among mitochondrial, MAM, and peroxisomal MAVS pools. Accordingly, it remains unclear whether PTMs themselves influence MAVS localization or whether organelle localization instead determines the repertoire and function of MAVS PTMs. Thus, an important future question is whether context-responsive PTMs, such as ASK1–p38-dependent phosphorylation induced by oxidative or ER stress, act uniformly across MAVS pools or preferentially modulate specific organelle-associated MAVS platforms. Another unresolved issue is how these spatially distinct MAVS pools are established. While the mitochondrial outer membrane insertase MTCH2 has been implicated in MAVS mitochondrial targeting (Guna et al., 2022), whether MTCH2 or other targeting pathways contribute to the establishment, maintenance, or PTM-dependent regulation of distinct MAVS pools remains largely unknown. Addressing these questions will provide a more integrated understanding of how organelle context and PTM-mediated regulation cooperate to determine MAVS signaling outputs.

Emerging evidence suggests that PTMs may contribute to this output selection by influencing not only signaling magnitude but also signaling fate. In this regard, MAVS carboxylation provides an example of a PTM-based switch that favors interferon induction while restraining apoptosis. Regulatory PTMs are distributed across multiple functional regions of MAVS, including the CARD and IDR, suggesting that the functional consequences of each modification are shaped by its structural position within MAVS.

This review provides a framework for understanding MAVS as an organelle-associated signaling hub in which metabolic and stress-related cues are translated into PTM-based regulation, while mitochondrial localization influences the functional consequences of these modifications. A key challenge moving forward will be to define the hierarchy and interplay among MAVS PTMs and to determine where output selection among IFN production, inflammatory signaling, and apoptosis is specified. Addressing these questions will be important for understanding how MAVS shapes antiviral defense, inflammatory signaling, and cell fate decisions in a context-dependent manner.
